# Effectiveness of Arnica and Bromelain for Improving Ecchymosis Following Facial Plastic Surgery: A Systematic Review

**DOI:** 10.1177/22925503261470270

**Published:** 2026-07-30

**Authors:** Kimya Manouchehri, Shaishav Datta, Marc Levin

**Affiliations:** 1 Department of Otolaryngology – Head and Neck Surgery, 7938 University of Toronto, Toronto, ON, Canada; 2 Division of Plastic, Reconstructive and Aesthetic Surgery, Department of Surgery, 12366 University of Toronto, Toronto, ON, Canada; 3 Lasky Clinic, Beverly Hills, CA, USA

**Keywords:** homeopathic remedies, arnica, bromelain, ecchymosis, edema, outcomes, arnica, bromélaïne, ecchymose, œdème, résultats, remèdes homéopathiques

## Abstract

**Introduction:**

Individual studies have suggested a benefit in using Arnica and Bromelain for reducing ecchymosis following facial plastic surgery; however, the literature remains inconsistent and methodologically heterogeneous. An up-to-date review is therefore necessary to synthesize the available evidence and provide clearer guidance to clinicians and patients alike. The objective of this study was therefore to investigate the effectiveness of Arnica and Bromelain in reducing postoperative ecchymosis after facial plastic surgery.

**Methods:**

A PRISMA-guided search of Medline, Embase, Cochrane databases, Epistemonikos, and ClinicalTrials.gov was conducted up to August 2025. Eligible studies included randomized controlled trials (RCTs) and observational studies evaluating Arnica and/or Bromelain use in facial plastic surgeries. The primary outcome was postoperative ecchymosis. Secondary outcomes included edema, adverse effects, and patient satisfaction.

**Results:**

Ten articles were included, comprising 6 RCTs, 3 prospective, and one retrospective cohort study, with a total of 696 patients (mean age 35 years, range 15-78). Most studies examined rhinoplasty (60%), followed by blepharoplasty (30%). Most studies (80%) evaluated Arnica use, of which 63% demonstrated significant reductions in postoperative ecchymosis at various time points. Edema was assessed in 2 Arnica studies, both of which showed significant reductions. Two studies evaluated Bromelain, with one demonstrating reduced ecchymosis by postoperative day 7. Adverse events were rare and mild.

**Conclusion:**

Arnica may help reduce postoperative ecchymosis and edema after facial plastic surgery; however, the evidence for Bromelain is limited. Marked heterogeneity in dosing and assessment tools limits the generalizability of findings and the ability to perform meta-analysis.

## Résumé

**Introduction:**

Certaines études ont suggéré un bénéfice de l’utilisation de l’arnica et de la bromélaïne pour réduire les ecchymoses après une chirurgie plastique faciale; cependant, la littérature reste incohérente et hétérogène sur le plan méthodologique. Une revue actualisée est donc nécessaire pour synthétiser les données disponibles et fournir des recommandations plus claires tant aux cliniciens qu’aux patients. L’objectif de cette étude était donc d’évaluer l’efficacité de l’arnica et de la bromélaïne dans la réduction des ecchymoses postopératoires après une chirurgie plastique faciale.

**Méthodes:**

Une recherche guidée par les critères PRISMA a été effectuée dans les bases de données Medline, Embase, Cochrane, Epistemonikos et ClinicalTrials.gov jusqu’en août 2025. Les études éligibles comprenaient des essais contrôlés randomisés (ECR) et des études observationnelles évaluant l’utilisation de l’arnica et/ou de la bromélaïne dans le cadre de chirurgies plastiques faciales. Le critère de jugement principal était l’ecchymose postopératoire. Les critères de jugement secondaires comprenaient l’œdème, les effets indésirables et la satisfaction des patients.

**Résultats:**

Dix articles ont été retenus, comprenant six ECR, trois études de cohorte prospectives et une étude de cohorte rétrospective, portant sur un total de 696 patients (âge moyen de 35 ans, intervalle de 15 à 78 ans). La plupart des études portaient sur la rhinoplastie (60 %), suivie de la blépharoplastie (30 %). La plupart des études (80 %) ont évalué l’utilisation de l’arnica; parmi celles-ci, 63 % ont démontré une réduction significative de l’ecchymose postopératoire à différents moments. L’œdème a été évalué dans deux études sur l’arnica, qui ont toutes deux montré des réductions significatives. Deux études ont évalué la bromélaïne, dont une a démontré une réduction de l’ecchymose dès le 7e jour postopératoire. Les événements indésirables étaient rares et bénins.

**Conclusion:**

L’arnica pourrait contribuer à réduire les ecchymoses et l’œdème postopératoires après une chirurgie plastique faciale; toutefois, les données concernant la bromélaïne sont limitées. Une hétérogénéité marquée au niveau des posologies et des outils d’évaluation limite la généralisation des résultats et la possibilité de réaliser une méta-analyse.

## Introduction

In facial plastic surgery, postoperative ecchymosis is a common finding that can negatively impact patient satisfaction and quality of life and is widely regarded as an undesirable sequela of surgery.^1,2^ Therefore, identifying treatments to reduce ecchymosis and improve healing is of significant interest to plastic surgeons and patients alike. Two agents that have gained attention are Arnica and Bromelain. Arnica, a botanical agent derived from the plant *Arnica montana,* has been used for centuries as a homeopathic remedy to alleviate pain, swelling, and bruising.^3,4^ Arnica is widely used and readily available over-the-counter; however, it is not approved by major regulatory authorities. Bromelain is a proteolytic enzyme complex extracted from the pineapple plant, *Ananas comosus,* and has been studied for its anti-inflammatory, antiedematous, and fibrinolytic properties, which may similarly aid in postoperative recovery.^5,6^

Several studies have evaluated Arnica and Bromelain for their potential to reduce postoperative ecchymosis, with several showing modest reductions in ecchymosis; however, the findings are inconsistent, and the overall quality of evidence is limited by methodological heterogeneity.^7‐12^ Additionally, prior systematic reviews have evaluated the perioperative use of Arnica and Bromelain across a broad range of surgical procedures; however, none, to our knowledge, focus specifically on their use for facial aesthetic surgeries.^2,12,13^ Therefore, the purpose of this systematic review is to specifically quantify the effectiveness of Arnica and Bromelain on reducing the severity of postoperative ecchymosis in these procedures. An up-to-date review is needed to clarify treatment effects and dosages, ultimately providing clearer guidance for clinicians and patients seeking evidence-based approaches to enhance recovery after facial plastic surgery.

## Methods

### Information Sources and Search

This review was performed in accordance with the guidelines outlined in the Preferred Reporting Items for Systematic Reviews and Meta-Analyses (PRISMA) statement.^14^ MEDLINE/PubMed, EMBASE, Cochrane Central Register of Controlled Trials (CENTRAL), Cochrane Database of Systematic Reviews, Epistemonikos, and ClinicalTrials.gov were searched between the date of database inception and August 3, 2025. Supplementary search methods included reference tracking, relevant reviews, and included studies.

### Eligibility Criteria

This review included patients of any age who underwent facial plastic surgery, including rhinoplasty, facelift, genioplasty, otoplasty, blepharoplasty, brow or lip lift, buccal fat removal, or any other facial plastic surgery. Any setting, such as hospitals and outpatient or private clinics, was included. Eligible studies examined the administration of Arnica and/or Bromelain via any route of administration, pre- and/or postoperatively. Comparators consisted of placebo, no treatment, or standard care. Randomized controlled trials, prospective or retrospective cohort studies, and case–control studies that reported on clinical, functional, or cosmetic outcomes related to postoperative ecchymosis, edema, adverse effects, or patient satisfaction were included. Exclusion criteria encompassed case reports, narrative reviews, editorials, expert opinions, or commentaries; studies that do not report on ecchymosis; studies unable to be translated into English; and those with no full text available. No publication date limit was set.

### Study Selection and Data Extraction

All citations from the literature search were imported into Covidence and screened by 2 independent reviewers. Eligible full texts were then assessed, with disagreements resolved by a third reviewer. Relevant data were independently extracted into standardized Microsoft Excel tables, piloted initially on 2 studies, and cross-referenced to ensure accuracy.

### Outcomes and Statistical Analysis

The outcomes of interest were measured through scales, instruments, or surveys to assess the impact of a given intervention. The primary outcome was the degree and/or duration of ecchymosis. The secondary outcomes included edema, adverse effects, and patient satisfaction. Due to heterogeneity in outcome measures, treatment protocols, and time points of Arnica/Bromelain administration, a meta-analysis was not conducted. Instead, a structured narrative synthesis was performed.

### Assessment of Risk of Bias

The methodological quality of the included studies was assessed using the National Institute of Health's (NIH) Guidance for Assessing the Quality of Controlled Intervention Studies and the Quality Assessment Tool for Observational Cohorts tool.^15^

## Results

### Study Selection

**Figure 1.**
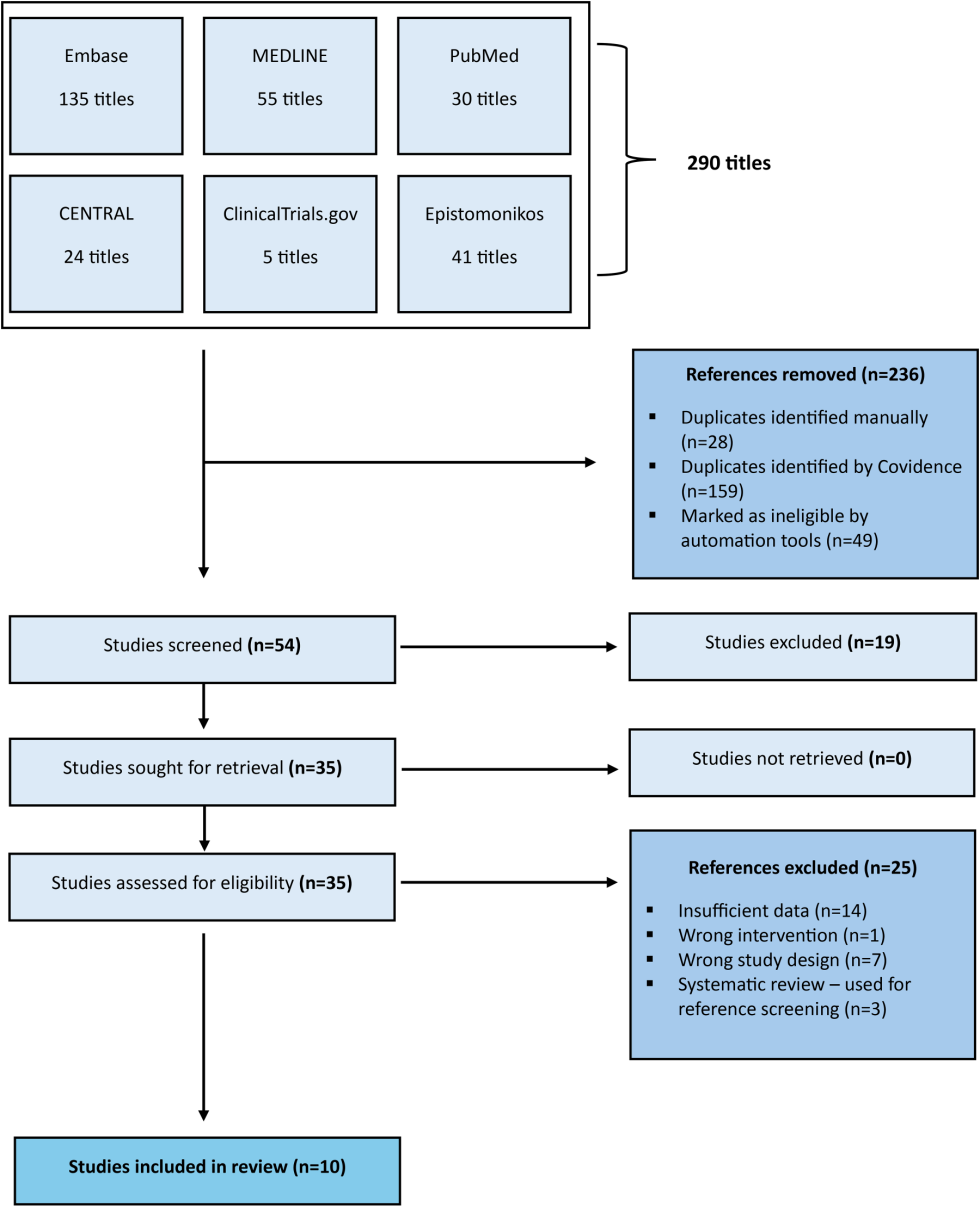
PRISMA flow diagram.

The literature search included a total of 290 articles. After removal of duplicates, 54 studies were screened by title and abstract, of which 35 proceeded to full-text review. Ultimately, 10 studies met the inclusion criteria and were included in the qualitative synthesis.^7,9-11,16-21^ Figure 1 outlines the PRISMA flow diagram for this review.

### Study Characteristics

Ten studies were included (Table 1).^7,9-11,16-21^ Of the 10 included studies, 6 (60%) were randomized controlled trials (RCTs),^7,10,11,16,19,21^ 3 (30%) were prospective cohort studies,^13,18,20^ and 1 was a retrospective cohort study.^17^ Publications spanned 1972 to 2025, with sample sizes from 22 to 155 patients (n = 696). Most studies were conducted in the United States (n = 5),^9,11,17,19,21^ followed by Turkey (n = 2).^10,18^ The majority (70%) were performed at tertiary care centres.^9,10,16-19,21^ Overall study quality was variable: 2 studies were rated as “good”,^7,16^ 7 as “fair”,^9-11,17-19,21^ and one as “poor” quality^20^ (Tables 2 and 3).

**Table 1.** Basic Characteristics of Included Studies.

#	Author, Year, and Country	Study Design and Time Frame	N (Groups)	Women, N (%)	Age in Years, Mean (Range)	Surgery Performed (%)	Intervention Type, Route, and Dosing	Timing and Frequency of Administration	Measured Outcomes
1	Totonchi and Guyuron, 2007, USA	RCT	Total = 48 16-IV dexamethasone and oral methyl-predinsone 16-Arnica 16-Control	37 (77.1)	NR (15-65)	Rhinoplasty (100)	Arnica; Oral; 3 × 500 mg 1M capsules, 9 × 500 mg 12C capsules (“C” = 100-fold serial dilution; and “M” = 1000-fold serial dilution)	Postoperative TID for 4 days 1M (500mg) capsule TID on POD0, then 12C (500mg) capsule TID on POD1-3	1-Extent of ecchymosis 2-Intensity of ecchymosis 3-Edema
2	van Exsel et al, 2016, Netherlands	RCT 01/2013 to 01/2014	Total = 116 59-Arnica 57-Placebo	88 (75.9)	55 (NR)	Blepharoplasty (100)	Arnica; Topical ointment; 10% ointment, contains 30g of *Arnica planta tota* mother tincture per 100g which is 10g of drug	Postoperative BID for 7 days	1-Subjective overall appearance of periorbital area 2-Degree of ecchymosis, erythema, edema, pain, and patient satisfaction, eyelid vertical aperture
3	Kang et al, 2017, USA	Retrospective cohort 07/2012 to 12/2012	Total = 27	16 (59.2)	NR (18-70)	Blepharoplasty (78.9), rhinoplasty (5.3), browpexy (5.3), cheekbone festoon and lower eyelid correction (5.3), levator advancement and/or ptosis repair (26.3)	Arnica; Topical hydrogel pads; Arnica 50M 50% and Ledum 50M hydrogel pads	Postoperative POD0-2 → continuous wear, change q6h POD3-6 → apply 3h per day	1-Healing (proxy for ecchymosis and edema combined)
4	Ozer Ozturk et al, 2024, Turkey	Prospective trial 10/2019 to 08/2020	Total = 115 26-Cold compresses 22-Cold and periorbital strip 21-Cold and Arnica 25-Cold and Reparil gel 21-Cold and Hirudoid	87 (75.6)	26.6 (NR)	Rhinoplasty (100)	Arnica; Topical cream; 5% Arnica cream	Postoperative TID application until ecchymosis resolved completely	1-Periorbital edema 2-Periorbital ecchymosis
5	Rahmaty et al, 2025, Iran	RCT	Total = 46 23-Bromelain 23-Placebo	38 (82.6)	31.5 (18-45)	Rhinoplasty (100)	Bromelain; Oral; 200mg tablets	Postoperative TID for 11 days	1-Ecchymosis 2-Edema 3-Subconjunctival hemorrhage
6	Kotlus et al, 2010, USA	RCT	Total = 30 15-Arnica 15-Placebo	0 (0)	NR (43-78)	Blepharoplasty (100)	Arnica; Oral; 3 × 500mg 1M capsules, 9 × 500mg 12C capsules (“C” = 100-fold serial dilution; and “M” = 1000-fold serial dilution)	Pre- and Postoperative TID for 4 days 1M (500mg) capsule TID (first dose before surgery) on POD0, then 12C (500mg) capsule TID on POD1-3	1-Degree of ecchymosis
7	Chaiet and Marcus, 2016, USA	Prospective trial 07/2010 to 06/2012	Total = 22 9-Arnica 13-Placebo	13 (59.1)	NR (18-59)	Rhinoplasty (100)	Arnica; Oral; 3 × 500mg 1M capsules, 9 × 500mg 12C capsules (“C” = 100-fold serial dilution; and “M” = 1000-fold serial dilution)	Pre- and Postoperative TID for 4 days 1M (500mg) capsule TID (first dose before surgery) on POD0, then 12C (500mg) capsule TID on POD1-3	1-Extent of ecchymosis
8	Simsek et al, 2016, Turkey	RCT 12/2013 to 12/2014	Total = 108 36-Arnica 36-Topical mucopolysaccharide polysulfate 36-Control	55 (50.9)	27.4 (NR)	Rhinoplasty (100)	Arnica; Topical; 75g	Postoperative QID for 10 days	1-Ecchymosis 2-Edema
9	Shehadi, 1972, Lebanon	Prospective trial	Total = 155 30-Control 32-Postoperative cooling (cold compress) 22-Operative and postoperative cooling 30-Bromelain 31-Antihistamines 10-Operative cooling and antihistamines	NR	NR (NR)	Rhinoplasty (100)	Bromelain; Oral; 50,000 Rorer units per tablet	Pre- and postoperative 2 tablets the night before surgery, 2 tablets the morning of surgery. 2 tablets QID on POD0-2, then 1 tablet QID for POD3-7	1-Ecchymosis 2-Edema 3-Subconjunctival hemorrhage
10	Seeley et al, 2006, USA	RCT	Total = 29 14-Arnica 15-Placebo	29 (100)	NR (NR)	Facelift (100)	Arnica; Oral; 3 × 500mg 1M capsules, 9 × 500mg 12C capsules (“C” = 100-fold serial dilution; and “M” = 1000-fold serial dilution)	Pre- and Postoperative TID for 4 days 1M (500mg) capsule TID (first dose before surgery) on POD0, then 12C (500mg) capsule TID on POD1-3	1-Degree of ecchymosis

Abbreviations: NR, not reported; BID, two times daily; TID, three times daily; QID, four times daily; POD, postoperative day.

**Table 2.** Methodological Quality of the Included Controlled Intervention Studies.

Criteria^a^ → Study ↓	1	2	3	4	5	6	7	8	9	10	11	12	13	14	Total^b^
1 (Totonchi and Guyuron^11^)	Y	NR	NR	Y	Y	Y	Y	Y	NR	Y	Y	NR	NR	NR	**Fair**
2 (van Exsel et al^16^)	Y	Y	Y	Y	Y	Y	N	Y	Y	Y	Y	Y	Y	N	**Good**
4 (Ozer Ozturk et al^18^)	N	NA	NA	NR	NR	Y	Y	NR	NR	Y	Y	NR	Y	NA	**Fair**
5 (Rahmaty et al^7)^	Y	Y	Y	Y	Y	Y	Y	Y	NR	Y	Y	Y	Y	Y	**Good**
6 (Kotlus et al^19^)	Y	Y	NR	Y	Y	NA	Y	NA	NR	Y	Y	NR	NR	NR	**Fair**
7 (Chaiet and Marcus^9^)	Y	Y	Y	Y	Y	Y	N	NR	Y	Y	Y	NR	Y	NR	**Fair**
8 (Simsek et al^10^)	Y	N	Y	N	Y	Y	NR	NR	NR	Y	Y	NR	NR	NR	**Fair**
9 (Shehadi^20^)	N	NA	NA	N	N	NR	Y	NR	NR	Y	Y	NR	Y	NR	**Poor**
10 (Seeley et al^21^)	Y	NR	NR	Y	NR	Y	Y	NR	NR	Y	Y	NR	Y	NR	**Fair**

**Criteria**:

1: Was the study described as randomized, a randomized trial, a randomized clinical trial, or an RCT?

2: Adequate method of randomization.

3: Concealed treatment allocation.

4: Blinding of study participants and providers.

5: Blinding of outcome assessors.

6: Similar groups at baseline on important characteristics affecting outcomes.

7: Overall drop-out rate of 20% or lower at study endpoint.

8: Differential drop-out rate of 15% or lower at study endpoint.

9: High adherence to the intervention protocols for each treatment group.

10: Other interventions avoided or similar in the groups.

11: Outcome measures clearly described, valid, reliable, and implemented consistently.

12: Report sample size with at least 80% power.

13: Prespecified outcomes reported, or subgroups analyzed.

14: Utilize an intention-to-treat analysis.

a Individual criteria are reported as “Y” (Yes), “N” (No), “NA” (Not applicable), or “NR” (not reported).

b Total quality is reported as “Poor,” “Fair,” or “Good.”

**Table 3.** Methodological Quality of the Included Observational Study.

Criteria^a^ → Study ↓	1	2	3	4	5	6	7	8	9	10	11	12	13	14	Total^b^
3 (Kang et al^17^)	Y	Y	NR	Y	N	Y	Y	N	Y	Y	N	N	Y	N	**Fair**

**Criteria**:

1: Was the research question clearly stated?

2: Was the study population clearly defined?

3: Was the participation rate of eligible persons at least 50%

4: Were all subjects selected or recruited from the same or similar populations? Were the inclusion and exclusion criteria for being in the study prespecified and applied uniformly to all participants?

5: Was a sample size justification, power description, or variance and effect estimate provided?

6: For the analyses, were the exposures of interest measured prior to the outcome being measured?

7: Was the timeframe sufficient so that one could reasonably expect to see an association between exposure and outcome?

8: Did the study examine the different levels of exposure as related to the outcome?

9: Were exposure measures clearly defined, valid, reliable, and implemented consistently across all participants?

10: Was the exposure assessed more than once over time?

11: Were the outcome measures clearly defined, valid, reliable, and implemented consistently?

12: Were outcome assessors blinded to the exposure status of the participants?

13: Was loss to follow-up after baseline 20% or less?

14: Adjustment for confounding variables?

a Individual criteria are reported as “Y” (Yes), “N” (No), “NA” (Not applicable), or “NR” (not reported).

b Total quality is reported as “Poor,” “Fair,” or “Good.”

Rhinoplasty was the most common procedure (n = 6),^7,9-11,18,20^ followed by blepharoplasty (n = 3),^16,17,19^ and one study assessing facelift.^21^ Among rhinoplasty studies, all patients underwent osteotomies.^7,9-11,18,20^ Ozer Ozturk et al,^18^ Chaiet and Marcus,^9^ and Simsek et al^10^ reported use of nasal splinting postoperatively for all patients. Similarly, Ozer Ozturk et al^18^ and Chaiet and Marcus^9^ reported the standard use of perioperative dexamethasone, whereas the remaining studies did not specify.^7,10,11,20^

### Patient Characteristics

Patient ages ranged from 15 to 78 years old, with an average age of 35. Four studies reported mean age for Arnica groups (34 years),^9,10,16,18^ and one for Bromelain (30 years).^7^ Across all studies, most patients were women (64.5%). Seeley et al^21^ only assessed women undergoing facelift who were described as nonsmokers and Caucasian, while Kotlus et al^19^ only assessed men. Few studies reported patient comorbidities.^7,18^ Ozer Ozturk et al^18^ reported that 2 patients (9.5%) in the Arnica group and 4 patients (15.4%) in the control group had systemic disease, with no significant difference between groups (*P* = .82). Rahmaty et al^7^ reported no patient comorbidities. Smoking status was disclosed in 3 studies.^16,18,21^ Seeley et al^21^ included only nonsmoking women in their study; Ozer Ozturk et al^18^ reported 3 patients (14.3%) in the Arnica group and 6 patients (23.1%) in the control group as smokers, with no significant difference between groups (*P* = .79). van Exsel et al^16^ had nearly equal proportions of smokers in their study, with 15 patients (25.4%) in the Arnica group, and 15 patients (26.3%) in the placebo group. Ozer Ozturk et al^18^ was the only study that reported alcohol use in patients and found no significant difference between groups (*P* = .69).

### Treatment Protocol

Eight studies evaluated Arnica administration,^9-11,16-19,21^ 2 evaluated Bromelain use,^7,20^ and none compared Arnica to Bromelain. Formulations included oral (n = 6)^7,9,11,19-21^ or topical (n = 4)^10,16-18^ administrations, with no studies comparing oral and topical formulations. Oral treatment protocols of Arnica^9,11,19,21^ all utilized the SinEcch (Alpine Pharmaceuticals) formulation, a 4-day regimen beginning on the day of surgery.^22^ The treatment protocol consists of three 500 mg 1 M capsules, where “M” corresponds to 1000-fold serial dilution, taken 3 times on the day of surgery. This is followed by nine 500 mg 12C capsules, where “C” corresponds to 100-fold serial dilution, taken 3 times daily for 3 days. The topical Arnica administration protocols,^10,16-18^ however, varied broadly in the concentrations and dosages (Table 1). In Bromelain studies, Rahmaty et al^7^ administered 200 mg oral tablets 3 times daily until POD (postoperative day) 11. Shehadi^20^ administered 2 Ananase tablets, each with 50,000 Rorer units, the night before surgery and every 6 h afterward for 5 days.

Totonchi and Guyuron^11^ studied the effectiveness of oral Arnica and systemic corticosteroids (10 mg IV dexamethasone intraoperatively followed by a 6-day oral methylprednisolone taper) separately on the extent of ecchymosis and edema postoperatively. Ozer Ozturk et al^20^ studied 5 groups, which included controls with cold compresses only, a periorbital strip group that had Steri-Strips applied along the inferior orbital rim, an Arnica 5% cream group, a Reparil gel group (1% aescin + 5% diethylamine salicylate), and a Hirudoid gel group (0.445% mucopolysaccharide polysulphate). Simsek et al^10^ studied both topical Arnica and mucopolysaccharide polysulphate cream to control patients, and Shehadi^20^ studied the effect of both oral Bromelain and antihistamines on controls.

### Effects of Interventions

All studies used patient photographs for assessment of postoperative ecchymosis and edema.^7,9-11,16-21^ Some included self-reported questionnaires.^16,17,19^ No single validated scoring system was utilized consistently across studies, and all studies had varying timepoints of outcome assessment postoperatively (Table 4).

**Table 4.** Ecchymosis and Edema Outcomes of Included Studies.

#	Author, Year, Country, Study Design	Surgery Performed (%)	Measurement Scale for Ecchymosis/Edema	Methods	Timepoints Measured	Significant Reduction in Ecchymosis Degree? (*P* Value)	Significant Reduction in Edema? (*P* Value)
1	Totonchi and Guyuron, 2007, USA RCT	Rhinoplasty (100)	Photographic scoring system (0-5 scale for ecchymosis extent, 0-4 scale for ecchymosis intensity, 0-3 scale for edema severity)	3 blinded panelists 3 groups: oral Arnica, control, corticosteroids	POD 2 POD 8	Extent/Intensity POD 2 (*P* = .19)/(*P* = .06) **POD 8 (*P* < .05)/(*P* < .01)**	**POD 2 (*P* < .0001)** POD 8 (*P* = .25)
2	van Exsel et al, 2016, Netherlands RCT	Blepharoplasty (100)	Photographic scoring system (photos rated as superior in appearance, similar, or worse)	6 blinded panelists (n = 3 medical, n = 3 nonmedical) 2 groups: topical Arnica and placebo	POD 3 POD 7 Week 6	Medical/Nonmedical Panel scores: POD 3 (*P* = .566/*P* = .690) POD 7 (*P* = .468/*P* = .815) Week 6 (*P* = 1.000/*P* = 1.000)	Medical/Nonmedical Panel *P* values POD 3 (*P* = .724/*P* = .586) POD 7 (*P* = .901/*P* = .853) Week 6 (*P* = 1.000/NA)
3	Kang et al, 2017, USA Retrospective	Blepharoplasty (78.9), rhinoplasty (5.3), browpexy (5.3), cheekbone festoon and lower eyelid correction (5.3), levator advancement and/or ptosis repair (26.3)	Physician-patient rating score with photographs rated as: A) markedly accelerated healing (7 days ahead of expected) B) accelerated healing (<7 days ahead of expected) C) no appreciable difference from expected	Unblinded (patients’ respective surgeons scored their healing) 2 groups: Arnica/Ledum hydrogel pads and control group	POD 1-2 POD 3-5 POD 6-8	**Markedly accelerated healing at POD 3-5, POD 6-8, and overall (*P*** = **.05 for all)**	
4	Ozer Ozturk et al, 2024, Turkey Prospective trial	Rhinoplasty (100)	Photographic scoring system (0-3 scale for edema and ecchymosis)	2 blinded observers 5 groups: cold application, periorbital strip, Arnica cream, Reparil gel, Hirudoid gel (Mucpolysaccharide polysulphate)	POD 1 POD 3 POD 5 POD 7 POD 10 POD 14 POD 21	POD 1 (*P* = .30) POD 3 (*P* = .66) POD 5 (*P* = .74) POD 7 (*P* = .45) POD 10 (*P* = .09) POD 14 (*P* = .26) POD 21 (*P* = 1.00)	POD 1 (*P* = .93) **POD 3 (*P*** = **.01)** POD 5 (*P* = .80) POD 7 (*P* = .46) POD 10 (*P* = .54) POD 14 (*P* = 1.00) POD 21 (*P* = 1.00)
5	Rahmaty et al, 2025, Iran RCT	Rhinoplasty (100)	Photographic scoring system: -Ecchymosis: score for % ecchymosis in each eye quadrant (none, <1/4, 1/4-1/2, 1/2-3/4, >3/4) -Edema: qualitative score for eye closure (none, minimal, partial, complete, full closure) Scoring done for each eye separately	1 blinded Otolaryngologist 2 groups: Bromelain tablets and placebo	POD 1 POD 3 POD 7 POD 11	Left/Right Eye POD 1 (*P* = .258)/(*P* = .665) POD 3 (*P* = .414)/(*P* = .228) **POD 7 (*P*** = **.012)/(*P*** = **.012)** POD 11 (*P* = .285)/(*P* = .285)	Left/Right Eyes POD 1 (*P* = .434)/(*P* = .570) POD 3 (*P* = .563)/(*P* = .651) POD 7 (*P* = .757)/(*P* = .757) POD 11 (*P* = .235)/(*P* = .235)
6	Kotlus et al, 2010, USA RCT	Blepharoplasty (100)	Photographic scoring system for the area (cm^2^) of detectable upper eyelid ecchymosis Rank order of all photos from least to most ecchymosis for POD 3 and 7 separately	1 blinded observer 2 groups: oral Arnica and placebo	POD 3 POD 7	Area (cm^2^)/Rank order of severity POD3 (*P* = .926)/(*P* = .854) POD7 (*P* = .267)/(*P* = .419)	NR
7	Chaiet and Marcus, 2016, USA Prospective trial	Rhinoplasty (100)	Photographic quantitative analysis: -Extent: area (cm^2^) circumscribed in Adobe Photoshop -Color change intensity: photos converted to CMYK mode (cyan, magenta, yellow, black), ecchymosis area quantified on a 0-255 scale	Blinded observer (unclear how many) 2 groups: oral Arnica and placebo	POD 2/3 POD 7 POD 9/10	Extent/Color change intensity * POD 2/3 (*P* = .468)/(*P* = .567) **POD 7 (*P*** = **.097**)/(*P* = .649) **POD 9/10** (*P* = .486)/**(*P*** = **.074)**	NR
8	Simsek et al, 2016, Turkey RCT	Rhinoplasty (100)	Photographic scoring system -Ecchymosis scale of 0-4 (0 = no ecchymosis; 1 = medial 1/3 of upper and/or lower eyelids; 2 = medial 1/2 of upper and/or lower eyelids; 3 = entire upper and/or lower eyelids; 4 = entire upper and/or lower eyelids and/or conjunctiva) -Edema scale of 0-4 (0 = no edema; 1 = mild edema; 2 = moderate edema; 3 = severe edema; 4 = complete edema)	2 blinded observers (study authors) 3 groups: Arnica cream, mucopolysaccharide polysulphate cream, control	POD 1 POD 2 POD 5 POD 7 POD 10	All Groups **POD 1 (*P*** = **.001)** POD 2 (*P* = .280) **POD 5 (*P*** = **.025)** **POD 7 (*P*** = **.007)** POD 10 (*P* = .364) Arnica vs Control **POD 1 (*P*** = **.006)** **POD 5 (*P*** = **.019)** **POD7 (*P*** = **.005)** Arnica vs mucopolysaccharide POD 1 (*P* = .374) POD 5 (*P* = .833) POD 7 (*P* = .694)	All Groups **POD 1 (*P*** = **.016)** POD 2 (*P* = .065) **POD 5 (*P*** = **.009)** **POD 7 (*P*** = **.005)** POD 10 (NA) Arnica vs control POD 1 (*P* = .011) POD 5 (*P* = .006) POD7 (*P* = .003) Arnica vs mucopolysaccharide POD 1 (*P* = .874) POD 5 (*P* = .974) POD 7 (*P* = .499)
9	Shehadi, 1972, Lebanon Prospective trial	Rhinoplasty (100)	Photographic scoring scale -Ecchymosis of eyelid scale of 1-4 (1 = light pink color, patchy distribution; 2 = light pink color, involving all of eyelid uniformly; 3 = bluish color and involving all of eyelid; 4 = dark blue or black color) -Edema of eyelid scale of 1-4 (1 = minimal swelling with skin creases visible; 2 = moderate swelling with skin creases effaced; 3 = marked swelling, eyelids could be opened actively; 4 = extreme swelling, eyelids could not be opened actively)	Unblinded observer 6 groups: control, cold compress postoperatively, cold compress intraoperatively and postoperatively, Ananase (Bromelain) group, antihistamines group, operative cold compress and antihistamine group	POD 1 POD 2 POD 5 POD 7 POD 14	No *P*-values reported No significant decrease in ecchymosis with Bromelain use (no raw values reported)	No *P-*values reported No significant difference in the intensity of edema with Bromelain use (no raw values reported)
10	Seeley et al, 2006, USA RCT	Facelift (100)	Photographic quantitative analysis: -Extent: area (cm^2^) circumscribed in Adobe Photoshop -Color change intensity: photos converted to CMYK mode (cyan, magenta, yellow, black), ecchymosis area quantified on a 0-255 scale	2 blinded observers (RN and MD) 2 groups: oral Arnica and placebo	POD 1 POD 5 POD 7 POD 10	Color change intensity/Area (cm^2^) differences **POD 1** (*P* = .09)/**(*P*** = **.005)** POD 5 (*P* = .16)/(*P* = .19) **POD 7** (*P* = .79)/**(*P* < .001)** POD 10 (*P* = .47)/(*P* = .13)	NR

Abbreviation: NR, not reported; POD, postoperative day; RCT, randomized controlled trial.

*P < .1 is significant. Bolded text indicates significance.

## Effectiveness of Arnica on Postoperative Ecchymosis and Edema

### Oral Regimens

Totonchi and Guyuron^11^ compared oral Arnica with corticosteroids and no intervention postrhinoplasty. On POD 2, the Arnica group had significantly reduced edema compared to control (*P* < .0001); however, it showed no significant difference in the extent or intensity of ecchymosis at this same time point. By POD 8, there was no significant difference in edema outcomes between groups. The authors concluded that Arnica may be effective in reducing edema postoperatively, particularly early on. In Kotlus et al,^19^ the authors used the same oral Arnica regimen in upper eyelid blepharoplasty. Ecchymosis was evaluated on POD 3 and 7, and no significant difference was found in the surface area or ranked severity of ecchymosis (*P* > .05). Seeley et al^21^ evaluated the effect of oral Arnica on bruising after facelift using both subjective data with a visual analog scale completed by patients and staff, as well as a novel computer system to quantify the color change intensity of ecchymosis (see Table 4 for more details). While the study found no significant difference in subjective reports of ecchymosis or color change intensity measurements, patients in the Arnica group had a significantly smaller area of ecchymosis (cm^2^) at POD 1 (*P* = .005) and POD 7 (*P* < .001). Chaiet and Marcus^7^ similarly found that oral Arnica use postrhinoplasty significantly decreased the extent of ecchymosis on POD 7 (*P* = .097, significance threshold set at *P* < .1) and color-change intensity by POD 9/10 (*P* = .074).

### Topical Regimens

In van Exsel et al,^16^ each patient had one eyelid randomized to 10% Arnica ointment and the contralateral eyelid to placebo ointment postblepharoplasty, with no significant alteration identified in periorbital appearance, ecchymosis, or edema relative to placebo. Kang et al^17^ retrospectively studied the use of topical Arnica hydrogel pads containing Ledum 50 M in reducing postoperative ecchymosis and edema after oculofacial surgery. In this study, the proportion of patients with markedly accelerated healing was found to be significantly greater than those with no appreciable difference at several time points (POD 3-5 and overall *P* = .05). In the 5-arm prospective trial by Ozer Ozturk et al,^18^ 2 blinded observers rated postoperative edema and ecchymosis of patients postrhinoplasty using a scoring system developed by Kara and Gokalan.^23^^,^^24^ There was no statistically significant reduction in ecchymosis between the groups (*P* > .05), although edema was significantly lower at POD 3 (*P* = .01), which the authors attributed to both Arnica and periorbital strip application. Simsek et al^10^ found that both topical Arnica significantly accelerated the regression of edema and ecchymosis on POD 2, 5, and 7 after rhinoplasty (*P* < .005). Taken together, topical Arnica demonstrated moderate benefit for edema and ecchymosis, but magnitude and durability of effect varied by study design.

### Effectiveness of Bromelain on Postoperative Ecchymosis and Edema

Two studies examined Bromelain. In a randomized, double-blind, placebo-controlled trial, Rahmaty et al^7^ found that Bromelain significantly reduced ecchymosis after rhinoplasty on POD 7 (*P* = .012) but did not affect edema. In contrast, Shehadi's^20^ earlier investigation comparing Bromelain, antihistamines, and cold compresses reported only a minor, nonsignificant improvement in ecchymosis with Bromelain, while the cold compress and antihistamine groups produced more pronounced effects.

### Assessment of Postoperative Satisfaction and Adverse Events

A few studies investigated patient satisfaction and adverse events. van Exsel et al^16^ found no difference in patient satisfaction at either POD 3 or 7 between placebo and Arnica ointment groups (*P* > .05). Similarly, Seeley et al^21^ found no significant group differences in patient satisfaction (*P* > .05). Patients in Kotlus et al^19^ were asked to report which eyelid (Arnica vs placebo) had the smoother recovery after blepharoplasty, with no significant differences found (*P* > .05). Adverse events were uncommon and mild. Chaiet and Marcus^9^ had one participant in the Arnica group who experienced a mild, self-limited rash which resolved within the study period.

## Discussion

This review demonstrates modest evidence supporting Arnica in particular for reducing postoperative ecchymosis and edema in facial plastic surgery. Across 10 studies and 696 patients, several trials suggested improvements in ecchymosis and edema.^7,9,10,17,18,21^ However, the ability to perform a meta-analysis was limited by variable outcome assessments and study designs. Given that visible ecchymosis of the face and neck has a disproportionate psychosocial impact, even small reductions can meaningfully improve patient satisfaction. Therefore, these findings are clinically relevant and should be considered by plastic surgeons when optimizing patient healing postoperatively.

Despite variation in topical Arnica regimens,^10,16-18^ this review can summarize the oral Arnica regimen as three 500 mg 1 M tablets followed by nine 12C capsules taken 3 times daily for 4 days.^9,11,19,21^ Two studies used a novel computer system for quantifying ecchymosis based on color intensity with Adobe Photoshop,^9,21^ which provided more objective measurements compared with the often subjective scales used. Seeley et al^21^ was one such study utilizing the computer system for quantifying color changes and found that patients taking Arnica postfacelift had significantly reduced ecchymosis at POD 1 (*P* = .005) and 7 (*P* < .001). Using the same system, Chaiet and Marcus^9^ concluded that patients taking Arnica postrhinoplasty had reduced ecchymosis at POD 7 (*P* = .097) and POD 9/10 (*P* = .074); however, their determination of significance was a *P*-value of <.1, thus limiting conclusions. Nevertheless, these significant results highlight the utility of computer-based models for measuring ecchymosis more objectively. Evidence for Bromelain, in contrast, was limited to 2 rhinoplasty studies. Rahmaty et al^7^ demonstrated significant reductions in ecchymosis severity, whereas Shehadi's^20^ earlier work showed only minor, nonsignificant effects.

Only 3 studies evaluated patient satisfaction, none demonstrating a significant difference compared with placebo.^16,19,21^ This likely reflects the multifactorial nature of postoperative satisfaction, influenced by comfort, cosmesis, and psychosocial readiness.^2^ Furthermore, these findings may reflect the challenges of long-term follow-up in aesthetic populations, as patients undergoing elective procedures may be lost to follow-up, especially if they recover uneventfully.^25^ Therefore, consistent, extended follow-up is needed to more accurately assess satisfaction outcomes.

Arnica is believed to exert anti-inflammatory and antioxidant effects by downregulating pro-inflammatory cytokines and reducing reactive oxygen species (ROS) within inflamed cells.^26^ These actions may decrease capillary leakage, limit local swelling, and accelerate resorption of extravasated blood to reduce ecchymosis. Bromelain's fibrinolytic and anti-inflammatory activity may similarly reduce local inflammation, vascular permeability, and tissue edema.^27^ However, the degree to which these mechanisms translate to clinical benefit is uncertain. Findings in this review generally align with studies from other surgical fields, such as dental and trauma surgery.^28-31^ In the setting of traumatic injuries and arthritis, a review by Toma et al^29^ found support for Arnica in treating ecchymosis and edema. Prospective and randomized trials on third molar extraction suggest that Arnica and Bromelain may reduce postoperative ecchymosis and edema, though the effect sizes are small and clinical significance is uncertain.^28,30,31^ Across all surgeries, a systematic review by Ho et al^12^ revealed inconsistent results, with only some RCTs showing improvement in ecchymosis and/or edema with Arnica (4/13) and Bromelain (5/7). The more pronounced effect of Bromelain in this review may be explained by the heterogeneity of included procedures, which encompassed molar extractions,^31^ orthognathic surgery,^32^ facial trauma,^33^ and episiotomies.^34^ Overall, most studies on this topic are centered on visible areas of the body, such as the face, neck, and hands,^35^ which reflects that visible bruising and swelling are of the most relevance to patient satisfaction, social recovery, and perceived procedural success.

Several limitations exist among the included studies. Kang et al^17^ utilized a retrospective study design with historical photographic controls. The assessment of healing was based on categorical ratings and by unblinded surgeons, introducing subjectivity and risk of observer bias. Additionally, this study utilized an intervention that combined Arnica and the homeopathic compound of Ledum, limiting the ability to isolate the effectiveness of Arnica itself. Chaiet and Marcus^9^ was the only study that used *P* < .1 for statistical significance, reportedly to account for the variability of skin pigment, extent of osteotomies, and history of nasal bone trauma. Notably, none of their results had a *P*-value of <.05. In Shehadi,^20^ no significant difference was found in the reduction of ecchymosis in patients receiving Bromelain; however, there was no mention of any attempt to conceal group allocation, and grading was performed by the same investigator and surgeon. This absence of blinding and randomization introduces performance and detection bias in this study.

**Figure 2.**
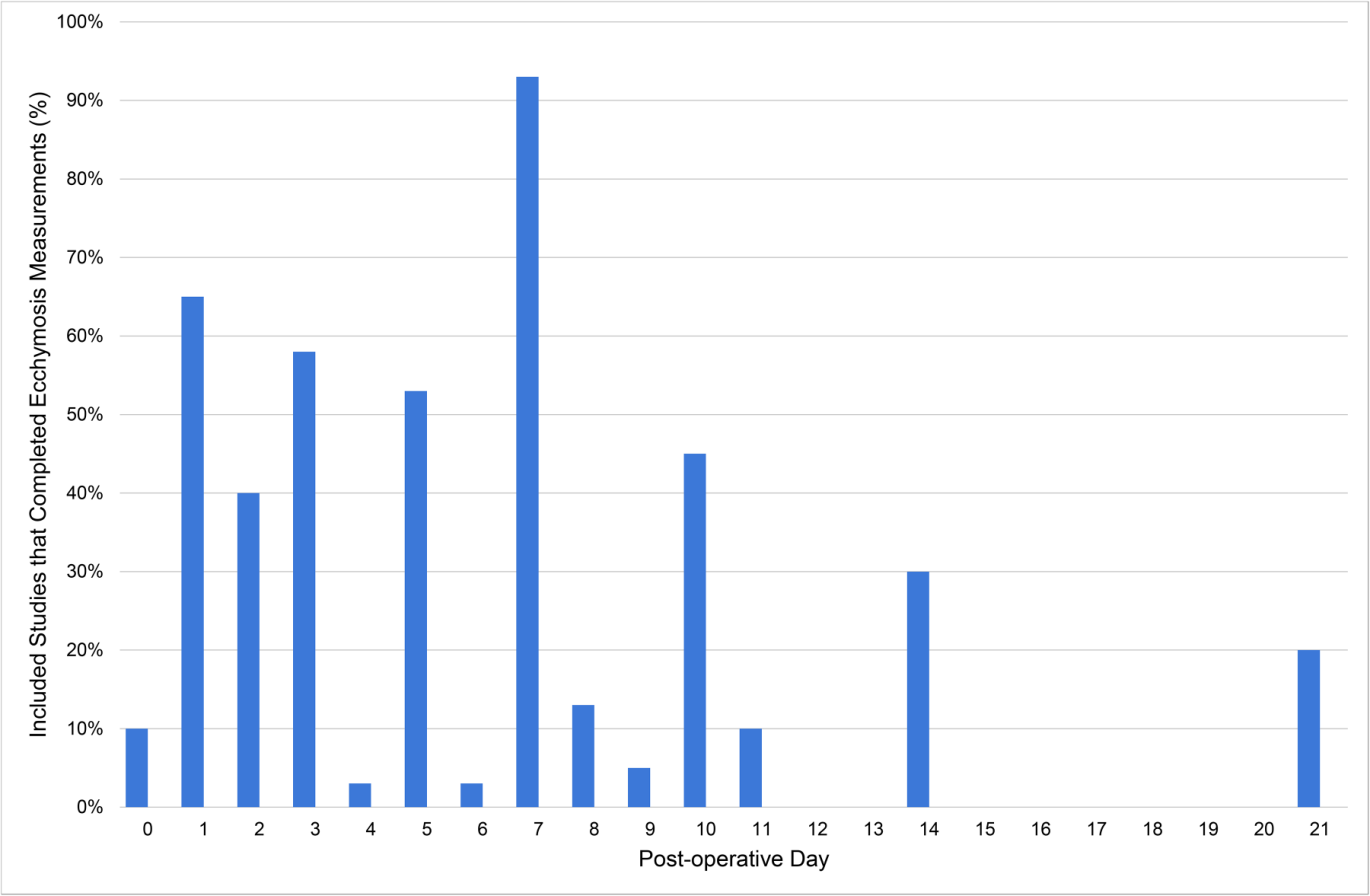
Timing of post-operative ecchymosis assessments.

The limitations of this review include the small number of eligible studies, most studies being of “fair” methodological quality, and the inability to perform meta-analysis due to design and measurement heterogeneity. Additionally, most studies were conducted in the United States, relied on smaller samples, and had variable timing of ecchymosis measurements (Figure 2), which restricts comparisons and conclusions on the progression of ecchymosis. Outcome assessments relied mostly on unvalidated and subjective scales, and some studies utilized unblinded raters. Finally, short follow-up windows further constrain clinical interpretations.

Future research should focus on standardizing treatment protocols and comparing oral to topical formulations, particularly with volumetric and colorimetric tools for ecchymosis measurement. Obtaining objective measurements of ecchymosis and edema is useful in personalizing postoperative management for patients undergoing plastic surgery. This review adds to the body of evidence that deems Arnica and Bromelain to have low toxicity, especially for short-term use, highlighting the opportunity for future clinical trials to more definitively study their efficacy under standardized conditions. Finally, long-term follow-up studies are needed to evaluate whether early reductions in ecchymosis meaningfully translate into improved satisfaction over time.

This review presents the most up-to-date synthesis of the available literature on Arnica and Bromelain in facial plastic surgery. While several randomized and prospective studies suggest modest reductions in ecchymosis and edema with Arnica in particular, evidence strength is limited by variable protocols and subjective outcomes. These findings underscore the need for further high-quality studies, particularly using oral Arnica regimens and volumetric or colorimetric tools to increase the reproducibility of findings. Given that ecchymosis can impact patient satisfaction and confidence, furthering our understanding of remedies to enhance healing will improve patient outcomes and experiences after facial aesthetic surgery.^36^
